# Contraception, punishment and women who use drugs

**DOI:** 10.1186/1472-6874-14-5

**Published:** 2014-01-09

**Authors:** Anna Olsen, Cathy Banwell, Annie Madden

**Affiliations:** 1 The Kirby Institute, The University of New South Wales, Sydney, Australia; 2 National Centre for Epidemiology and Population Health, The Australian National University, Canberra, Australia; 3 The Australian Injecting and Illicit Drug Users League (AIVL), Canberra, Australia

## Abstract

**Background:**

In light of the recent debate on the use of financial incentives to promote long-acting contraception and sterilisation among women who use illicit drugs we discuss attitudes to contraception, pregnancy and parenting among Australian women who inject drugs.

**Methods:**

Qualitative interviews were conducted with 90 women of reproductive age about contraceptive use, preferences, reproductive histories, attitudes to and experiences of parenting. All women were either currently, or had previously injected drugs. The in-depth, semi-structured interviews were compared and contrasted for themes relating to drug use, contraception, pregnancy and parenting.

**Results:**

Participants aspired to control their fertility, expressed individual contraceptive preferences and concerns for their children (both born and unborn). Most had tried a number of contraceptive methods interspersed by periods of non-use related to experiences of side-effects, being single or abstinent, believing that they were infertile and trying to conceive. Attitudes varied from woman to woman and in the same individual over their life course. Some believed that they were not likely to be capable, but most aspired to be successful mothers.

**Conclusions:**

Women’s drug use should not automatically be associated with an inability to make informed health care choices or to care for children. Evidence suggests that women who use drugs do not need to be paid to limit or end their fertility. Rather, programs that aim to reduce barriers to obtaining free, non-discriminating reproductive advice and parenting assistance would better utilise women’s agency to improve their own reproductive health.

## Background

Periodically, public alarm is raised about the potential negative impact of women’s drug use on children’s health and well-being. This occurred recently in Australia driven by the possible introduction of a controversial privately funded program, Project Prevention [1]. Operating in the USA and the UK the program pays women who use alcohol and/or other drugs US$300 (UK£200) to be sterilised or take long-acting contraception. While the program addresses men and women who use alcohol or other drugs, its emphasis is on women. According to public statements (see http://www.projectprevention.org/) the aims of Project Prevention are 1) to prevent or reduce the number of children born that are affected by drugs and 2) to prevent women from having children who are deemed to be unable to care for them because of their drug use. The program’s underlying rationale is that people who use alcohol and other drugs are both incapable of making appropriate decisions about their fertility and caring for children.

In a recent visit to Australia the program’s founder, Barbara Harris, told the Age newspaper that if funding is obtained she expects to bring the program to Australia (http://www.theage.com.au/national/charity-preys-on-weak-addicted-20101019-16sjp.html). No details were provided on its proposed format, but it is likely that if it were introduced, it would replicate the US program in which participants provide evidence of their long–term drug use and medical evidence of the sterilization or long-term contraception implantation before receiving payment.

Objectors to Project Prevention have criticised the program for isolating a specific population of people, especially low-income, minority women, for fertility control and contravening basic human rights [2-7]. As the program offers a single service – reproductive control to people who use drugs – without aims to assist or improve other health or social needs such as dependency, long-term economic insecurity and homelessness, Project Prevention has been compared with eugenic movements. In the social sciences the aims and objectives of Project Prevention have been used to explore neoliberal discourses of female drug users as ‘risky’ reproductive agents and the neo-liberal governing of (pregnant) bodies by state and non-state agencies [8].

With respect to incentivising health behaviours, there is increasing interest in international development, public health, and clinical medicine as to whether cash payments or other economic incentives can be used to influence the choices and behaviours of individuals and groups in order to promote desired health goals [9]. Some evidence suggests that prizes and other incentives can encourage people to make short-term behavioural changes such as smoking cessation [10]. However, experts have also noted the need to address the potential for the coercive effect of incentives on patient autonomy. Many objectors to Project Prevention have argued that using comparatively large financial gifts to discourage economically disadvantaged people who use drugs from having children is morally and ethically inappropriate [2-4,6,11,12].

Programs such as Project Prevention draw attention to the complex and, in some cases, unresolved health and social issues regarding women’s illicit drug use, reproduction and parenting. Evidence for poor reproductive health outcomes among women who inject drugs is varied. Unintended pregnancies are often reported to be high [13] as well as high pregnancy rates and high rates of adverse pregnancy outcomes (miscarriage, termination and stillbirth) [14]. However, these findings are often not compared with national averages where unintended pregnancy and termination rates in the general population are also considered high [15,16].

Clinical data suggests that heroin use during pregnancy contributes to poor maternal and child outcomes and causes an increase in obstetric complications [17] as well as neonatal complications [18,19]. However, meta-analyses have shown that social disadvantage and tobacco use are often not considered in analyses [20,21]. Such potential confounders are rarely considered for their possible contributions to poor neonatal outcomes.

As reflected in the aims of Project Prevention, concern about the health and well-being of children whose parents use drugs is widespread. Children of people who use drugs are considered to be at risk of neglect and abuse [22,23] and parental drug use is increasingly seen as a factor in child protective service provision [23,24]. It is, however, acknowledged that the nature of any relationships between substance use and child-rearing are complex, and that poverty and psychological disorders are contributing factors to poor child outcomes [25].

Challenging simplistic cause and effect associations, a small body of literature cautions against conflating parental drug use with child neglect [26]. In particular, sociological research shows that mothers who use drugs often feel capable as parents, place a high value on motherhood and hold strong ideals around childrearing [27-29]. Further, barriers to fertility control, rather than a lack of agency in making health decisions, may explain poor health outcomes in this population [30].

In terms of Australia’s response to drug use, policies are guided by a harm minimisation framework. Rather than explicitly punitive approaches, specialised drug support and pre and post natal services are available for the 1.3% to 2% of women who use illicit drugs during pregnancies in Australia [23,31]. Pregnancy is seen as an ideal time to involve women in the health care system and, despite prejudice displayed by some health care professionals towards people who use drugs [32-34], pregnant women can be highly motivated to engage in health care provision such as opioid substitution therapy [35]. Stable use of opioids, such as methadone maintenance, during pregnancy reduces maternal illicit opiate use and fetal exposure, enhances interaction with obstetrical care, and is associated with improved neonatal outcomes like heavier birth weight [17,18,36].

Many (but not all) contraceptive methods and brands are available on the Australian Pharmaceutical Benefits Scheme, making them low cost for women eligible for government assistance. Yet, little is known about contraceptive use among Australian women who inject drugs. Surveys suggest that they have a lower level of contraception use than the general population. Approximately 60% of all Australian women aged 18 to 44 use contraception [37]. Lower levels have been reported among women who use drugs – between 34% [38] and 54.7% [14] – yet this survey data may not be useful in describing the intentions of a particular woman at any point her reproductive life. Behaviours around sex and pregnancy intendedness are emotional, inter-personal and contextual and cannot be easily measured by surveys and scales [39-41]. In other words, survey data should be interpreted cautiously when applied to population level health data and interventions.

In light of interest in Project Prevention in Australia and conflicting evidence on the reproductive health choices of women who use drugs we seek to provide an in-depth and nuanced picture of contraceptive use, pregnancy and mothering by Australian women with a history of injecting drug use. We draw on empirical evidence to discuss whether the underlying assumptions made by Project Prevention about women who use drugs are valid and whether the program would be appropriate to the Australian context. Elucidating women’s attitudes and experiences, we discuss health care priorities for this population and their children.

## Methods

During 2005–06 in-depth, semi-structured interviews were conducted with 109 women who were currently injecting drugs or had done so in the past, and who were hepatitis C positive. Data from a sub-sample of women of reproductive age, aged 45 years or younger (n = 90), have been extracted for this paper. We employed a purposive sampling framework in order to represent women from a range of socio-economic backgrounds. We recruited interviewees from community organisations such as HCV councils, peer-based organisations for people who inject drugs, drug treatment services, and health clinics in two urban cities in Australia: Canberra and Melbourne. Ethical approval was received from the Australian National University Human Research Ethics Committee and written consent was received from all participants.

A brief, standardized set of questions was used to collect socio-demographic data followed by open-ended questions on past and current contraceptive experiences, drug use histories, reproductive and sexual health, hepatitis C related health, current living arrangements, and future plans. Interviews were digitally recorded and professionally transcribed. Qualitative data were entered into Atlas.ti [42].

Preliminary analysis of the qualitative data was guided by themes from the research literature and interview questions related to reproduction, contraception, hepatitis C and drug use. Specific experiences within these topics were compared and contrasted while new themes such as poverty, housing and concerns about children emerged. For each theme identified, transcripts were re-read and re-coded, systematically comparing interviews in order to build links between themes and condense any that overlapped. The research team jointly reflected on the conduct and content of interviews. Pseudonyms are used throughout.

As this is a relatively large qualitative study across two metropolitan cities in Australia we believe that the findings are relevant to other Australian women who inject drugs. While we find similarities between this data and findings from research conducted internationally, the different country approaches to health care provision and policy means that direct comparison may not be valid. One potential limitation of our Australian findings is that women were recruited from health care centres, hepatitis C councils and peer-based organisations for people who inject drugs suggesting that they already seek and receive health care. It is not clear whether their experiences differ from women who do not use these services; it is possible that women who are not in regular contact with health services use contraceptives less and hold different attitudes to drug use and child bearing.

## Results

The primary illicit drug of use amongst participants was heroin, although amphetamine, benzodiazepine and other opiate injection was also reported. Most women were disadvantaged with achieved education levels of Year 10 or lower and an annual household income of less than $20,000 per annum (See Table 1). Women frequently described struggling to make ends meet between welfare income and payments for housing, food, children’s items, and drugs.

**Table 1:** Socio-demographic characteristics

	**N = 90**	
**Age in years**	**Range 17-45**	**Mean 32**
	**N**	**%**
**Education**		
Attended tertiary education	8	8
Completed Yr 11 + 12	25	28
**Employment**		
Employed part-time, full-time, casual	14	15
**Relationship status**		
Single, separated, widowed, divorced	35	39
In a relationship, de facto, long-term boyfriend, married	55	61
**Household income**		
<$20,000	73	81
**Assets**		
Receiving benefits	82	91
Owns a car	25	28
Owns a home	6	7
**Children**		
Number of women with at least one child living with them	68	75
**Justice system**		
Ever remanded in custody or jailed	35	39
**Drug use**		
Injected drugs in the last 12 months	83	92

Contraception use by 90 women of reproductive age is shown in Table 2. Over half of the women (60%) reported that they did not use contraception in the last 12 months, five of whom were pregnant or were trying to conceive. Twelve women were using long-acting contraceptive methods. Several women reported using condoms as well as other contraception.

**Table 2:** Contraception used in the past 12 months

	**N = 90**
**None**	
No contraceptive use (includes abstinence, withdrawal, celibacy)	49
Pregnant or trying to get pregnant	5
**Permanent methods**	
Tubal ligation, partner vasectomy	6
**Long-acting methods**	
Implanon	6
Depo	5
IUD	1
**Reversible methods**	
The pill	4
Condoms	17*
Diaphragm	0

*Condoms were sometimes used in conjunction with other contraceptives.

### Contraceptive use

The paid provision of contraception by Project Prevention is limited to a small number of permanent or long-acting methods currently on the market (male and female sterilisation, intrauterine devices (IUDs) and Implanon). Easily reversible and user-controlled contraception (eg hormonal pill, NuvaRing, diaphragm, condoms and spermicides) are not supported by the program and the long-acting method Depo-provera is no longer supported. In order to elucidate women’s experiences of contraception and the implications a particular method can have on her life trajectory, we discuss their experiences with four categories of contraception: (1) permanent sterilisation (2) long-acting methods (3) easily reversible methods and (4) non-use.

### Sterilisation

Six women in our study reported having had a tubal ligation or that their male partner had had a vasectomy. Some women had resorted to tubal ligation early in their lives to escape the risk of pregnancy during a period of heavy drug use. Reflecting on her past drug use, Diane, who was in her mid-forties, illustrates the multiple contextual factors that many women take into account when considering this option. Her account contradicts the notion of the irresponsible drug-user and demonstrates her many efforts to manage her fertility.

I was twenty-one and I was pretty full on into dope. I was homeless. I was living in Sydney just at people’s places. I thought my life was not going to work out and I was always going to be an addict. Even though I didn’t want to be I just thought that’s the path I’m going and I can’t see any way out. I had terrible problems with IUD. I had rotten experiences with the pill. I didn’t trust condoms as a form of contraception and one of my mates said, ‘Have your tubes tied’, I just did.

### Long-acting methods

Most women in this sample had preferred to keep their reproductive options open. At the time of the interview 12 were using long-acting hormonal contraceptives (IUD, Implanon or Depo Provera). In addition to fertility control, these methods were chosen because women were not required to remember to take a daily tablet (the pill), and because of their side-effects of reduced or no menstruation. In contrast to sterilisation, long-acting methods also allowed for pregnancy in the future. For example, Rebecca, a 23 year old, chose Depo Provera because she aspired to a ‘normal’ life in the future that included a stable relationship and healthy and happy children.

I’d like to have a child but I want to do the right thing by the child too. I want to try and be the best parent I can be. I don’t mind if I don’t have the child until I’m 30 or however it works out. I might not even have children, it might not work out that way, but at the moment I don’t think I could look after a child to the best of my ability or to what the child deserves. So it’s better safe than to be sorry so that’s why I just get the injections (Depo Provera) instead of using a condom.

In the details of their reproductive histories many more women reported previously trying a long-acting hormonal contraceptive but ceasing when they wanted to get pregnant, ended a relationship, method failure or, commonly, when they experienced unpleasant side effects such as menstrual irregularities. Sam, a 30 year old, recounts how her attempt to responsibly manage her “lifestyle” was thwarted:

Yes, that [implant] was horrible. My sisters recommended it, they both had had it. Because I was living the [drug-using] lifestyle that I was I thought it would be a good idea to get the implant, but I kept bleeding all the time with it.

### Easily reversible methods

In our study four women were using the hormonal pill but most women had used it at one point of their reproductive lives. The hormonal pill was often one of the first methods of pregnancy prevention or was used to regulate menstruation. Women’s rationales for giving up the pill included: change in relationship status, side effects, to “give their body a break” from the synthetic hormones, or because of method failure (unplanned pregnancy).

For example, when Imogen, 30 years old, was younger she found that the pill caused her to menstruate continuously for six months and saw this as evidence of the dangers of contraception. The experience was linked to stopping that contraception and a subsequent unplanned pregnancy.

… and I had the pill, tablets when I was a teenager and I just bled the whole time … The whole six months I just bled. I couldn’t handle it and when I stopped taking the pill it was like I didn’t bleed for two months and then I was pregnant … It just stuffs around the body too much that kind of crap.

Seventeen participants reported using condoms, some in conjunction with other methods. Although, women generally talked about the condom as a method for preventing sexually transmissible infection (STI) rather than pregnancy. Condoms were often used during casual sex or at the beginning of a relationship when the couples’ STI status was unknown. Once an STI test had been performed, condom use became irregular or stopped within that relationship and was not always replaced by other contraceptives.

Clara, 33 years old, had experimented with several methods of contraception ending in intermittent condom use. Her contraceptive career was punctuated by unplanned pregnancies, two of which were terminated.

… *tried them all, actually, all. None of them work … I found I experimented with different contraception, like, the injection, the pill, and I had found that nothing had seemed to - I couldn’t take anything … The injection made me vomit continuously. Really badly. And the pill just made me feel sick all the time. So, when I started working at prostitution when I was about nineteen, so after that it was just condoms.*

### Non-use of contraception

Over half of the sample (60%) reported no current contraceptive use. Five of these women were pregnant or were trying to get pregnant. A few women blamed themselves for not being organised enough to use contraception while others provided logical reasons for not doing so including: not being able to find a method that they were comfortable using; concerns about the negative effects of contraception on long-term fertility; being abstinent or single; not expecting to have sex; or only having sex with women. As we have discussed elsewhere, some were disinterested in sex due either to previous abusive relationships or a lowered libido which can come with opiate use [43,44]. A few women considered their use of the withdrawal or the rhythm methods as contraception although these methods are not widely recognised as effective.

Many interviewees’ reproductive histories reflected intervals when they did not use contraception despite sexual activity and not wanting to get pregnant. A common reason provided for non-contraceptive use was the belief that they were infertile as a result of their drug use; a belief arising from their experience of amenorrhea or dysmenorrhea. As Vanessa, 29 years old, explains, women frequently understood lack of menstruation as infertility.

I was on the methadone programme last year for ten months and I didn’t get a period then. I thought that was a form of contraception but I was mistaken … I went to the hospital and they said [I was] pregnant and I was in shock. It was wrong timing. I was thinking of joining the army reserve and getting work. I had my life planned out for the next five years and then the pregnancy happened and that just threw everything out of whack. Now I have accepted and I am doing my best to make sure that I can provide for this child and that.

### Pregnancy & motherhood

Patterns of contraceptive use and attitudes to contraception were also intertwined with women’s experiences of, and hopes for, their fertility. Contraception could allow one to fulfil aspirations of being a responsible woman. However complete or satisfactory fertility control was regularly reported to be difficult to achieve and contraceptive needs could be overtaken by other aspects of their lives. Many women had experienced unplanned pregnancies and terminations, sometimes several. Yet the narratives women used to describe these experiences suggest more than hapless, out of control bodies.

They were, for example, aware of the potential damage to their children, born and unborn, of drug use and other aspects of their lives. They often commented negatively about combining drug use with pregnancy and parenthood and aimed to refrain from having children until they no longer used drugs or their use was more stable. Several younger women revealed plans for controlling their reproduction now, while aspirations of parenthood lay in the future. They discussed ideals of how and when they wanted to become a mother, what type of motherhood they aimed to realise as well as changes to their drug use and economic independence.

The prospect of motherhood (both planned and unplanned) provided some women with an incentive to stop using drugs or to go onto treatment. As Christine, a 28 year old single mother and a previous injecting drug user, described:

Because it really gave me the strength that I don’t know I would have had otherwise, to pull myself out of a pretty bad lifestyle, pretty bad head space, the whole situation that I was in for a while. It sort of gave me that understanding that some things are more important.

Others felt that they had successfully reconciled motherhood and drug dependency. They attempted to shield their children from any ill-effects of their drug use by setting aside money and hiding their drug use. Pam, 33 years old, a mother of two and expecting a third child made the point: *you can be a heroin addict and have kids - you get money the first thing you’ve got to do is make sure your kids are alright; make sure they’re fed, they’re warm and that*.

Similarly, Julianne, a 39 year old with a troubled history of drug use, sex work and domestic violence described the success and independence of her children.

… but my kids, they know everything and they all did Year Twelve and done all their studies and went to college … They have done all that at their young age and I am so proud of them. They didn't turn out like me. It wasn't in my blood to go and have drugs.

Still, many women were not in custody of their children who had been adopted out, were cared for by family members or had been removed by government services. Some women were trying to regain custody of their children, others had accepted the judgement that they were inadequate mothers for these children, or for any further children, because of their drug use. Children were usually removed involuntarily, although several women reported relinquishing care of their children when issues related to drug use became insurmountable. They perceived this agreed child removal as a form of good mothering, albeit undesirable and painful. Roseanne, a 27 year old in a de facto relationship, had recently placed her youngest child in foster care.

I was living on my own with my son. I had my son with me, and then I put him in foster care when he was fourteen months old. The biggest mistake I ever done, but it was the best thing for him anyway.

## Discussion

These descriptions of contraception, pregnancy and mothering are mixed and at times ambivalent. Despite this, interviewees’ voices express agency, purpose and hope and provide a nuanced picture of women’s attitudes and behaviours, and thus health care needs, of Australian women who inject drugs. Our analysis addresses, in the Australian context, the main concerns driving Project Prevention: the assumption that people who use drugs, particularly women, do not have the capacity, facility or agency to engage contraceptive methods without considerable incentive; and that they are not suitable parents.

This study demonstrates first that women who inject drugs are capable of organising permanent, long-acting and reversible contraception. Like women in the general population [37,45,46], women who inject drugs provided an array of reasons for stopping and starting various contraceptive methods at different times [43,44]. Some reported regular contraceptive use while most described a history in which they used one form of contraception after another interspersed by a lengthy hiatus. At different stages of their lives and drug histories women reported recognising that their ability to parent a child was threatened and drug use itself could also be a primary motivator for contraceptive use, or more exceptionally, sterilization.

Reasons for not using contraception at a particular time were diverse and meaningful and included: experience of side-effects, being single, believing that they were infertile, trying to conceive and being pregnant. Despite being highly sexualised in much of the literature, women also discussed abstinence, disinterest in sex and lowered libido [43,44] as reasons for non-use or irregular use of contraception. This data reveals a more nuanced picture of the sexual and reproductive behaviours of women who use drugs. Rather than out-of-control bodies, interviewees describe making choices and report many of the same reproductive experiences as other women bringing into question the underlying assumptions of Project Prevention and the appropriateness of the intervention in the Australian context.

Second, this research provides a more nuanced picture of pregnancy and parenting among women who use drugs than common risk-based analyses or survey research. Assumptions made about women’s parenting capabilities ignore the evidence that they can be capable of providing a nurturing and loving environment for their children [47,48] even in the midst of injecting drug use and economic insecurity [29,49-52]. Some women we interviewed had lost children to social services and expressed doubts about their capacity to parent. However, most women aspired to be successful parents and those caring for children described actively working to prevent harm to their children. This echoes other research which suggests that women can modify, decrease or cease their drug use in response to contextual factors, including children and resources [48,51] and shows that they can recognise when they need help with parenting [26].

Together, these findings highlight women’s agency in making health decisions [30] and bring into question a number of Project Prevention’s more-punishment based objectives. In particular, we suggest that there are alternative approaches to Projection Prevention that better fit within a harm reduction approach to illicit drug use. For example, free contraceptive advice and availability accepts that women who use drugs value motherhood, can plan pregnancy and value reproductive health information (as opposed to preventing their child-bearing through incentivisation).

Currently all contraceptive devices, other than condoms, are prescribed and/or inserted by health care professionals in Australia so any response to improving contraceptive up-take and reproductive advice for women who use drugs will require the availability of easily accessed, non-discriminatory services. Within the health care system, stigma and discrimination towards people who inject drugs is shown to negatively impact on their accesses to quality service provision [32,53,54]. Health services providing information and access to contraception must adhere to principles including informed consent, respect for each patient, bodily integrity and the right to refuse medical treatment [4,5] which do not easily fit with the objectives of Project Prevention.

Furthermore, our data suggest that emerging programs and policy discourse should be concerned with treatment and holistic care, rather than prevention of procreation. Currently Australians who use drugs report barriers to drug treatment access including long waiting times, lack of support and childcare concerns [55,56]. More broadly, many people who use illicit drugs in Australia experience disadvantage with respect to general health, co-morbidities and economic poverty [57,58]. Socio-economic difficulties experienced by parents and families [27,59], including the emotional and social impact of stigma, marginalisation and demoralisation [60-63] need to be considered alongside health care policy and practice.

Finally, there are practical and ethical differences between offering services that will pay for contraception, including sterilisation if the individual prefers this method, and paying women to control their fertility. Incentivised or openly coercive contraceptive schemes “embody the erroneous premise that low income women are not competent to make their own contraceptive decisions… only to strip low income women of their dignity and decisional autonomy” [4]. Particularly when it comes to drug use, pregnancy and parenting, “the mother’s needs disappear and they are only considered for the harms they transmit to their children” [27]. In targeting only the reproductive capacity of some women, Project Prevention fails to address women's needs for comprehensive reproductive health care, effective drug treatment programs, mental health services, and social, economic and educational support. Rather than fixing any particular social or population health problem, such openly targeted programs are ethically questionable, only serve to punish and further marginalise people who use drugs, especially women, [64] and fail to utilise their agency to improve their own health and that of their family.

## Conclusion

Empirical evidence suggests that women who inject drugs are capable of organising permanent, long-acting and reversible contraception and do not need to be paid to limit or end their fertility. Further, that parental drug use is not synonymous with child abuse. Broader critiques of the neo-liberal governing of pregnant bodies also highlight the ethical problems associated with targeting certain women for reproductive control. Rather, than punishing women for their drug use, health programs that aim to reduce barriers to obtaining free, non-discriminating reproductive advice and parenting assistance would better utilise women’s agency to improve their own reproductive health.

## Competing interests

The authors declare no completing interests.

## Authors’ contributions

AO and CB participated in the design of the study, data collection, analysis and drafting of the manuscript. AM participated in the drafting of the manuscript. All authors read and approved the final manuscript.

## Pre-publication history

The pre-publication history for this paper can be accessed here:

http://www.biomedcentral.com/1472-6874/14/5/prepub
